# Gamified interventions to educate healthcare professionals on the rational use of antimicrobials

**DOI:** 10.3389/fmicb.2025.1577005

**Published:** 2025-10-27

**Authors:** Mahadevaiah Neelambike Sumana, Supreeta R. Shettar, Yogeesh D. Maheshwarappa, G. K. Megha, Veerabhadraswamy G. S., Chinchana Shylaja Eshwarappa, Shruthi Shree S. C.

**Affiliations:** Department of Microbiology, JSS Medical College and Hospital, JSS Academy of Higher Education and Research, Mysuru, India

**Keywords:** gamified intervention, tackling AMR, rational use of antimicrobials, ICMR antimicrobial treatment guidelines 2022, antimicrobial stewardship (AMS)

## Abstract

**Background:**

Antimicrobial resistance [AMR] is a global health problem. It is important to train health care professionals on the rational use of antimicrobials to curb AMR.

**Methods:**

This prospective interventional study was conducted with clinical practitioners, undergraduates [MBBS/Interns], postgraduates and pharmacy Students. A total of 50 participants were included in the study. The innovative games were administered for the management of infections of all the different systems of the body under the Indian Council Medical Research (ICMR) treatment guidelines of 2022 and the latest Infectious Disease Society of America (IDSA) guidelines involving different components. Pre-test and post-test questionnaires were administered and evaluated.

**Results:**

After the intervention, the knowledge on the ability to differentiate between bacterial and viral symptoms in respiratory tract infections and gastroenteritis improved from 48 to 94%. The practice of using the right empirical choice of antimicrobials at the right dose for the right duration, on the basis of the severity of the infection, improved from 34 to 82%. The awareness/practice of using the right and rational combination of antibiotics improved from 44 to 84%. Knowledge of suspected multidrug-resistant gram-negative infections and other priority pathogens, such as methicillin-resistant *Staphylococcus aureus* [MRSA] and *Candida* infection, has improved from 32 to 78%. The practice of using certain antibiotics at specific infection sites based on the basis of their pharmacodynamics and pharmacokinetics improved from 20 to 76%. The knowledge of the intrinsic resistance of certain microorganisms to specific antimicrobial agents has improved from 15 to 80%.

**Conclusion:**

The gamified intervention successfully improved participants’ knowledge and awareness of rational antimicrobial use. The substantial improvements in all the aforementioned components highlight the positive impact of the intervention in promoting optimal antimicrobial use and curbing AMR. Innovative gamified interventions create better and long-lasting awareness, ensuring the appropriate use of antimicrobials.

## Introduction

Antimicrobial resistance [AMR] has become a serious global health problem. The World Health Organization [WHO] has identified AMR as one of the top ten public health concerns with the potential to kill 10 million people per year by 2050 (WHO policy guidance on integrated antimicrobial stewardship activities, 2023; Charani et al., 2021). Initially, penicillin revolutionized the treatment of infectious diseases. Healthcare and global development have transformed to reduce mortality. The continued introduction of various antimicrobial agents has enhanced healthcare, but their incorrect use at all levels of human, animal, and plant health has unleashed AMR repercussions (Jonas et al., 2017).

In the face of rising AMR, Lower middle income countries (LMICs) health and development policies aiming to eliminate severe poverty (living on less than $1.90 per day) by 2030 have proven impossible. According to the World Bank, the impact of AMR on healthcare expenses might vary from $300 billion to more than one trillion dollars by 2050 (Charani et al., 2021). In May 2015, the World Health Assembly established a global action plan for antibiotic resistance. This includes strategic objectives aimed at raising awareness and knowledge of antimicrobial resistance, as well as optimizing the use of these medications (WHO policy guidance on integrated antimicrobial stewardship activities, 2023). Continuous training is required both socially and professionally in various ways, such as through the use of media, conferences by specialists in the field, and antimicrobial optimization programs.

One of the most significant challenges in AMS programs is the amount of knowledge that students must master in a short period (Singh et al., 2021; Taneja and Sharma, 2019). AMR is a difficult concept to comprehend, and many people, including pharmacists and dentists, believe that the person develops resistance (Hsu, 2020; Woodhead and Finch, 2007). Up-to-date knowledge of optimal antimicrobial use appears to be quite poor in medical students and practitioners. Medical educators face a significant challenge in developing and implementing instructional approaches that promise to increase students’ knowledge of antimicrobial resistance and antimicrobial stewardship (Wutzke et al., 2007; Fuller et al., 2022; Mendelson et al., 2016).

Existing programs frequently exhibit mixed results in terms of effectiveness. The conventional method of teaching students [lectures] has not actively engaged the students. It is largely a passive strategy that has not been proven to increase long-term memory or understanding of AMR concepts. It has been suggested that games actively include participants in the AMR learning process, which helps address these constraints (Gautham et al., 2021; Azevedo et al., 2013).

Games have recently been increasingly employed in AMS programs, as games are more effective than conventional methods. Games can increase interest in a topic and reinforce previously provided information. They have been demonstrated to foster a competitive environment and boost student interaction. As a result, games could be viewed as a suitable supplement to existing healthcare initiatives, addressing the limitations of “conventional techniques” (Al-Amin et al., 2021; Collins et al., 2022). This study aimed to determine how games can increase knowledge about antibiotic prescription practices by creating an engaging learning environment.

## Methodology

This prospective interventional study was conducted with clinical practitioners, undergraduates [MBBS/Interns], postgraduate and pharmacy students of JSS Medical College and JSS College of Pharmacy, Mysuru, South India. A total of 50 participants were included in the study. The participants were divided into 2 teams. The pretest questionnaire was first administered to each participant over a period of 20 min. The intervention was then conducted as follows:

1. Introduction about rational use of antibiotics, the importance of antimicrobial stewardship interventions in a tertiary hospital setting.
2. Playing innovative games designed for the management of infections of different systems [based on the latest ICMR antimicrobial treatment guidelines 2022 and the latest IDSA treatment guidelines].
3. A post-test Questionnaire was administered to assess the effectiveness of the intervention.

### Questionnaire

A multiple-choice questionnaire covering all systemic infections was designed with 150 marks, with 30 questions, each with a weightage of 5 marks. The questions focused mainly on the empirical choice of the right antimicrobial agent at the right dose and duration for a particular infection, with antibiotics to be avoided under certain conditions on the basis of pharmacokinetics and pharmacodynamics and intrinsic resistance. The participants answered the questionnaire independently for over 20 min without using reference materials, notes, or assistance. Thus, baseline knowledge was evaluated.

### Game design

The innovative games were designed for the management of infections of all the different systems of the body under the ICMR-AMR treatment guidelines of 2022 and the latest IDSA guidelines, which focus on the following:

1. To differentiate between bacterial and viral features in common infections, such as respiratory tract infections and gastroenteritis, as most often these infections are caused by viruses, they are treated with antibiotics.
2. To understand the right empirical choice of antimicrobials at the right dose for the right duration based on the severity of infection.
3. To use the right and rational combination of antibiotics.
4. To suspect multidrug-resistant gram-negative infections and other priority pathogens causing infections, such as Methicillin-resistant *Staphylococcus aureus* (MRSA) and Candida.
5. To highlight antibiotics that are not useful at certain infection sites based on the pharmacodynamics and pharmacokinetics of the antibiotics.
6. Intrinsic resistance of some organisms to antimicrobial agents.
7. Miscellaneous- Dosage calculation based on renal functions, rational choice of investigations at different stages of infection, and choosing the right antibiotic based on the breakpoint minimum inhibitory concentration quotient [BMQ].

Table 1 highlights the innovative use of interactive games to enhance understanding of rational antimicrobial use. Each game is designed to target specific learning objectives, from differentiating bacterial and viral infections to selecting the right antibiotic, dosage, and duration.

**Table 1:** Innovative games for rational antimicrobial use.

Objective	Games Used
Differentiating bacterial vs. viral infections	Basketball, Bucketing the Ball, Monkeying with Donkey
Selecting the right antibiotic	Carom, Dart, Hopscotch, Where in Venn, Musical Chair
Teaching antibiotic combination therapy	Death by Double Strike
Selecting antimicrobials based on severity	Hierarchy by Severity, Build Your Burj Khalifa, Where in Venn
Selecting antimicrobials based on site of infection	Drop the Dropout, Push to the Pandora
Understanding correct dosage & duration	Fun with Numbers, Bowling Game, Statue Game, Pick Your Partner Right

Table 2 presents a gamified educational strategy for rational antimicrobial use across various infections. Each game is designed to reinforce key learning objectives, such as differentiating bacterial and viral infections, selecting appropriate antibiotics based on severity and site, and understanding optimal dosage and duration.

**Table 2:** Innovative games for rational antimicrobial use across various infections.

Infection type	Games used	Educational objective
Respiratory tract infections	Basketing the Ball	Differentiate bacterial vs. viral pharyngitis, sinusitis, and LRTI to reduce unnecessary antibiotic use
	Fun with Numbers	Select the right dose and duration for bacterial pharyngitis and LRTI
	Bowling Game	Select the right antibiotic, dose, and duration for bacterial sinusitis
	Death by Double Strike	Select the appropriate antibiotics for CAP treatment
	Hierarchy Game	Select the right antibiotics for LRTI based on severity
	Push to Pandora	Understand antibiotics to avoid in respiratory tract infections during pregnancy
	Dart Game	Identify the correct antibiotics and those to be avoided in LRTI
Gastrointestinal & intra-abdominal infections	Monkeying with Donkey	Segregate bacterial vs. viral gastroenteritis to prevent unnecessary antibiotic use
	Statue Game	Select the right antibiotic, dose, and duration for bacillary dysentery, cholera, amoebiasis, and giardiasis
	Build Your Burj Khalifa	Select antibiotics for mild, moderate, and severe intra-abdominal infections to ensure appropriate escalation
	Paint Your Imagination	Learn antimicrobial options for liver abscess, cholecystitis, pancreatic abscess/necrosis, with or without risk of candidiasis
Urinary tract infections	Picture Puzzle	Treatment options for uncomplicated cystitis
	Pneumonic (AIM-PE, P-TIMES, COLD)	Treatment options for pyelonephritis, prostatitis, and Epididymo-orchitis
	Drop the Dropout to Pandora	Identify antibiotics to avoid in UTIs
	Fun with Numbers	Understand the dosage and duration of empirical antibiotics for uncomplicated cystitis and pyelonephritis
Acute undifferentiated fever	Island and Woodland Game	Recognize clinical features supporting AUF
	The Loop Game	Identify the causative agents of fever with jaundice, sore throat, and rash in AUF
	Where in Venn	Select appropriate antibiotics for enteric fever, rickettsial fever, and leptospirosis
	Push to Pandora	Rational selection of laboratory investigations in AUF
Sepsis & MDRO infections	Tall and Short Doctors Race	Understand sepsis and septic shock management
	Picture Puzzle	Learn to suspect MDRO infections
	Story Creation	MRSA suspicion and treatment
	Pneumonic	Suspicion and treatment of Pseudomonas and Candida infections
Skin & soft tissue infections (SSTIs)	Dart Game	Identify antibiotics that reduce toxin production
	Scrabble	Learn treatment options for SSTIs
	Bowling Game	Antibiotics that do not act on anaerobes and antibiotics with antitubercular activity
	Color Your Imagination	Learn necrotizing fasciitis treatment
	Picture Puzzle, Where in Venn	Select treatment for deep neck space infections
Central nervous system (CNS) infections	Story-Based Game	Recognize diagnostic pointers for acute febrile encephalopathy/acute encephalitis syndrome
	Where in Venn	Learn treatment options for meningitis

### Statistical analysis

Data analysis was carried out using SPSS software version 20.0. The effectiveness of the intervention was evaluated by comparing pre-test and post-test scores across six components among 50 participants. Confidence intervals for the mean percentages were calculated at a 95% confidence level. A two-tailed z-test for proportions was used to determine the statistical significance of the observed differences.

## Results

The various components of rational antimicrobial use (as outlined in the methodology) were assessed pre-test and post-test.

### Pre-test result analysis

The knowledge on intrinsic resistance of certain microorganisms to specific antimicrobial agents [Component VI] was the least during the pre-test [15%]. The knowledge on suspecting multidrug-resistant gram-negative infections and other priority pathogens, such as Methicillin-resistant *Staphylococcus aureus* [MRSA] and *Candida* [Component IV], was also very low [32%]. The practice of using the right empirical choice of antibiotics for the right duration based on the severity of the infection [Component II] was satisfactory [34%]. The practice of using certain antibiotics in specific infection sites based on their pharmacodynamics and pharmacokinetics [Component V] was better when compared to the other five components [20%]. The awareness/practice of using the right and rational combination of antibiotics [Component III] was also better when compared to the other five components [44%]. The knowledge about differentiating between bacterial and viral symptoms in common infections like respiratory tract infections and gastroenteritis [Component I] was the best when compared to the other five components [48%].

### Post-test result analysis

After the intervention, the knowledge about differentiating between bacterial and viral symptoms in common infections like respiratory tract infections and gastroenteritis improved from 48 to 94%. The practice of using the right empirical choice of antimicrobials in the right dose for the right duration, based on the severity of the infection, improved from 34 to 82%. The awareness/practice of using the right and rational combination of antibiotics improved from 44 to 84%. The knowledge on suspecting multidrug-resistant Gram-negative infections and other priority pathogens, such as Methicillin-resistant *Staphylococcus aureus* [MRSA] and *Candida* infection, improved from 32 to 78%. The practice of using certain antibiotics in specific infection sites based on their pharmacodynamics and pharmacokinetics improved from 20 to 76%. The knowledge of the intrinsic resistance of certain microorganisms to specific antimicrobial agents improved from 15 to 80%. Table 3 depicts the comparison between pre- and post-test results of six different components on the rational use of antibiotics.

**Table 3:** Depicts the comparison between pre- and post-test results of six different components on the rational use of antibiotics.

Component	Pre-test in %	Pre-test CI	Post-test in %	Post-test CI	*p* value
I	48	34.15 to 61.85	94	87.42 to 100.58	4.10 × 10^−9^
II	34	20.87 to 47.13	82	71.35 to 92.65	2.62 × ^10–8^
III	44	30.24 to 57.76	84	73.84 to 94.16	4.57 × 10^−6^
IV	32	19.07 to 44.93	78	66.52 to 89.48	1.85 × 10^−7^
V	20	8.91 to 31.09	76	64.16 to 87.84	1.31 × 10^−11^
VI	15	5.10 to 24.90	80	68.91 to 91.09	<10^−15^

## Discussion

The findings of this study show that a gamified intervention is an effective strategy for improving knowledge and awareness of the rational use of antimicrobials. Observed improvements across all six components suggest that gamified educational strategies can significantly influence participants’ comprehension of complex antimicrobial stewardship principles. Using gamification as a teaching tool engages learners and makes learning interactive, which is particularly important for addressing antimicrobial resistance (AMR), a global health challenge. This approach allows learners to actively participate in scenarios that mimic real-life decision-making, fostering greater comprehension of the topic.

The intervention enabled participants to distinguish bacterial symptoms from viral symptoms in common infections, highlighting how gamified methods can deliver targeted education. This study demonstrated an improvement from 48 to 94%, resonating with findings reported by Azevedo et al. (2013). They also demonstrated that educational activities significantly improved knowledge regarding the correct use of antibiotics for bacterial infections rather than viral infections. According to Gyssens (2018), gamified educational strategies were more effective than traditional methods in improving healthcare professionals’ knowledge of AMR-related concepts. These results highlight gamification’s ability to equip individuals with the knowledge needed to make informed choices about antimicrobial use, thereby reducing inappropriate antibiotic prescriptions.

Another impact of the intervention was the improvement in participants’ understanding of antimicrobial selection based on the severity of the infection. Gamified platforms, as highlighted by Gorbanev et al. (2018), are valuable for teaching clinical decision-making. Gamification simulates real-life scenarios, enhancing learners’ ability to apply theoretical knowledge to practice (Gorbanev et al., 2018). This process bridges the gap between education and clinical application. The intervention also improved awareness of rational antibiotic combinations by emphasizing synergy and minimizing resistance development. Previous studies, such as Molina et al. (2020), have shown that interactive and gamified educational settings significantly improve participants’ comprehension of pharmacological principles, making the learning process more impactful.

In the context of AMR, the intervention’s focus on detecting multidrug-resistant pathogens was particularly notable. Calik et al. (2022) demonstrated that gamified interventions helped learners diagnose and prepare to address drug-resistant infections. This improvement translates into better infection control practices and more targeted antimicrobial therapy, ultimately improving patient outcomes. Similarly, a better understanding of the pharmacodynamics and pharmacokinetics of antibiotics for site-specific infections highlights the intervention’s success in imparting critical knowledge for effective treatment. Zainuddin et al. (2020) noted that gamification fosters dynamic educational settings that facilitate the retention of complex information, supporting the findings of this study.

The improved understanding of certain organisms’ intrinsic resistance to specific antimicrobials demonstrates the intervention’s substantial impact. This understanding is crucial for combating AMR by avoiding irrational antimicrobial use. Nowbuth et al. (2023) emphasized the role of gamification in antimicrobial stewardship education, demonstrating its ability to transform knowledge into practice by reinforcing key stewardship principles through engaging and memorable experiences.

Gamified interventions in healthcare education are increasingly recognized for their relevance and efficacy. This study contributes to the growing body of literature by addressing the limitations of traditional didactic approaches, which often fail to sustain learners’ interest or promote active participation. Contemporary educational strategies prioritize interactive and experiential learning, and gamified interventions align perfectly with these priorities.

The gamified approach adopted in this study is a significant step forward in antimicrobial stewardship education, offering a replicable model for broader implementation. As Mansen (2023) suggested, integrating gamification into healthcare education not only improves knowledge retention but also cultivates critical thinking and problem-solving skills. The results of this study strongly indicate that gamified interventions can be scaled across varied clinical and educational settings, contributing meaningfully to the global fight against AMR.

## Conclusion

The gamified interventions employed in this study successfully improved participants’ knowledge and awareness of the rational use of antimicrobials. These improvements are a step ahead in curbing AMR and optimizing patient outcomes by reducing unnecessary antimicrobial prescriptions. Continued efforts to enhance the knowledge and create awareness on the rational use of antimicrobials are the need of the hour for combating the global challenge of AMR and preserving the efficacy of the antimicrobial agents for future generations. Innovative gamified interventions create better and long-lasting awareness, promoting rational use of antimicrobials to curb the growing AMR.

## Data Availability

The original contributions presented in the study are included in the article/supplementary material. Further inquiries can be directed to the corresponding author.
